# Simian Virus 40 Large T Antigen as a Model to Test the Efficacy of Flouroquinolones against Viral Helicases

**DOI:** 10.6026/97320630014075

**Published:** 2018-02-28

**Authors:** Sammer Siddiqui, Muhammad F. Anwar, Sadaf Naeem, Syed Hani Abidi, Shamshad Zarina, Syed Ali

**Affiliations:** 1 Department of Comparative Pathology, Tulane University, New Orleans, LA, USA; 2 National Center for Proteomics, University of Karachi, Karachi, Pakistan; 3 Department of Biochemistry, University of Karachi, Karachi, Pakistan; 4 Department of Biological and Biomedical Sciences, Aga Khan University, Karachi, Pakistan; 5 Department of Pathology, Dow University of Health Sciences, Karachi, Pakistan; 6 Department of Biological Sciences, Nazarbayev University School of Medicine, Nazarbayev University, Astana, Kazakhstan

**Keywords:** SV40 LT-Ag, Fluoroquinolones, Antiviral drugs

## Abstract

Simian virus 40 large T-antigen (SV40 LT-Ag) is a 708 amino acid nuclear phosphoprotein. Among many functions of LT-Ag is its ability to perform as an ATPase-helicase, catalyzing the unwinding of viral genome during replication. The LT-Ag has been employed in the studies of helicase structure and function, and has served as a model helicase for the screening of antiviral drugs that target viral helicase. In this study, using in vitro enzyme assays and in silico computer modeling, we screened a batch of 18 fluoroquinolones to assess their potential as antivirals by virtue of their inhibition of the LT-Ag helicase. We found all fluoroquinolones to be inhibitory to the helicase activity of LT-Ag. In our docking analysis, most of these tested drugs showed similarity in their interactions with LT-Ag. Our study shows the potential of fluoroquinolones as antiviral drugs and of SV40 LT-Ag as a model protein for screening drugs against viral helicases.

## Background

Simian virus 40 (SV40) is a small, non-enveloped DNA virus [1]. The virus' genome comprises two sets of genes: late and early expressed [2]. The late expressed genes encode structural proteins necessary for viral assembly; these include three capsid proteins VP1, VP2, VP3 and a maturation protein agnoprotein [2]. The early expressed genes encode proteins required for viral replication. This set includes the small and large T antigens (STAg and LT-Ag, respectively) [3]. The SV40 LT-Ag is a 708 amino acid nuclear phosphoprotein, with multiple biochemical activities [4]. The LT-Ag is able to transform many cell types; its transforming ability is mainly dependent upon its interaction with cellular factors including retinoblastoma and p53 tumor suppressor proteins [3]. The LT-Ag also acts as a regulator of viral and cellular gene expression, and as an initiator of viral DNA replication [5, 6]. The LT-Ag binds to specific sites in the region of SV40 replication origin and promotes the local unwinding of DNA. LT-Ag recruits cellular DNA replication proteins to the site, which include Topoisomerase I, replication protein A, and DNA polymerases, and thus promotes SV40 DNA replication [7].

Among many functions of LT-Ag is also its ability to perform as an ATPase-helicase, catalyzing the unwinding of viral genome during replication, harvesting the energy required for this process by means of ATP hydrolysis [6]. The SV40 LT-Ag is a member of superfamily 3 of helicases, which consists of ring forming helicases [7]. In addition to oncology research, LT-Ag has been employed in the studies of helicase structure and function. The LT-Ag has served as a model helicase also for the screening of antiviral drugs that target viral helicases [8]. In our previous study, LT-Ag was used as a model helicase to develop a non-radioactive helicase assay [9]. For the current study, using in vitro and in silico methods, we screened a batch of 18 fluoroquinolones to assess their potential as antivirals by virtue of their binding to, and inhibition of, the LT-Ag helicase.

## Methodology

### Helicase Assay

Purified SV40 LT-Ag was purchased from CHIMERx, USA. As described previously [9], the helicase substrate was prepared by mixing single strands of complimentary oligonucleotides in 1:1 ratio to a final concentration of 20 μM each, heated to 95°C for 5 min, and then allowed to anneal by gradual cooling to room temperature for 1 hr. Typically, each helicase reaction contained 20μl 5x helicase buffer (200mM Tris pH-7.6, 25mM MgCl2, 20mM DTT, 125mM KCl, 10% glycerol and 0.5μg/ulBSA), 1mM ATP, 10μM DNA oligo substrate, 1x SYBR Green I and 1.44μM purified helicase, in a total volume of 100μl. The reaction was incubated at 37°C for 30 min and carried out in triplicates. Adding 4μl of 0.5M EDTA stopped reaction. Fluorescence was measured using Chameleon Fluorescence Reader 2 (Hidex, Finland), at excitation/emission 492/535 nm. For calculating the helicase activity, readings were corrected by subtracting the background fluorescence of a SYBR Green-only blank reaction mixture from the fluorescence of the sample. The enzyme activity was then expressed as percentage of unwound substrate as follows:

Percentage of unwound substrate = [(y-x)/y]*100

Where x and y represent the fluorescence of reaction, respectively, with and without any added helicase.

### Enzyme Inhibition Assay

Inhibition assays for helicase activity were performed as reported previously [9, 10]. Eighteen fluoroquinolones, in concentrations of 0.1, 1, 10 or 100 μM, were tested for their inhibition on LT-Ag helicase activity. These tested fluoroquinolones were: Levofloxacin, Ciprofloxacin, Ofloxacin, Balofloxacin, Fleroxacin, Enoxacin, 8Hydroxquinolinol, Pefloxacin, Lomefloxacin, Nalidixic acid, Enrofloxacin, Cinoxacin, Difloxacin, Flumiquine, 8Quinolinol, Sparfloxacin, Norfloxacin, and Moxifloxacin. The results were expressed as percent inhibition observed in the presence of the inhibitor, taking LT-Ag helicase activity as 100% in the absence of any added inhibitor.

### Docking Studies

To analyze the interactions between LT-Ag and the selected fluoroquinolones, crystal structure of SV40 LT-Ag (PDB ID: 1SVO) was obtained from Protein Data Bank (http://www.rcsb.org), while the SDF files of the fluoroquinolones were obtained from the Pubchem library of the NCBI database (http://www.ncbi.nlm.nih.gov/pccompound). The SDF files were subsequently converted to mol2 format by using OpenBabel GUI [11]. The binding pocket for the drug was predicted using Molegro Virtual Docker (MVD) software. To select the helicase-binding site, coordinates of the binding sphere were designated between Glu473 and Lys445, whereas the radius of search sphere was kept at default setting, i.e., 10Å. To predict protein-ligand interactions, Lead IT or FlexX version 2.0, software was employed [12, 13]. The docking was performed using the default parameters of the program, and the Single Interaction Scan algorithm of Lead IT was used for this analysis. This software generates more than 100 docking poses and presents them in ascending order with respect to docking energy. To verify these results, the same analyses were also performed with MVD software.

## Results

### Inhibition of LT-Ag Helicase Activity by Fluoroquinolones

**Figure 1:**
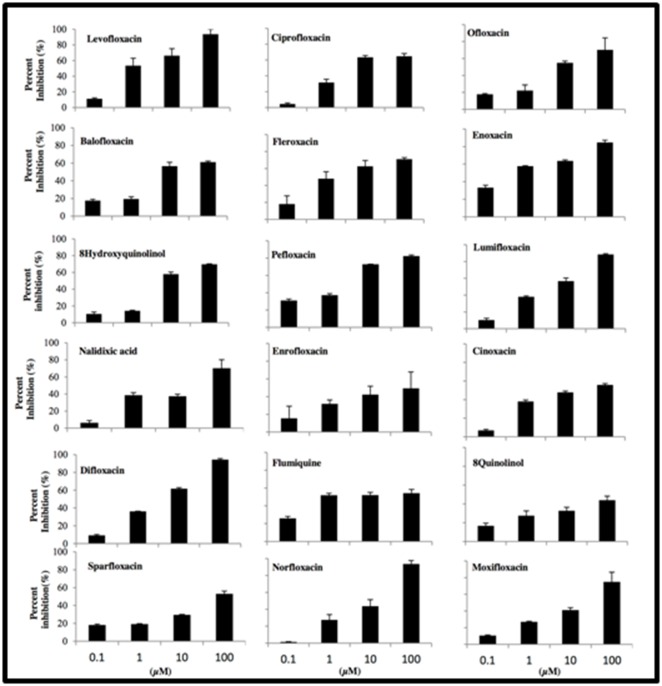
LT-Ag helicase inhibition by fluoroquinolones: Bars represent percent inhibition of LT-Ag helicase activity treated with 0.01, 0.1 μM, 1.0 or 10μM of the fluoroquinolone. With few exceptions, most of the fluoroquinolones exhibited 30-70% inhibition of LTAg helicase activity at 1.0μM and 10μM concentration. Each bar represents mean of triplicates ± SEM.

The LT-Ag helicase activity was measured in the presence of 0.1, 1.0,10 and 100μM fluoroquinolone. Percent inhibition of LT-Ag helicase activity by all 18 fluoroquinolones was observed to follow almost a linear trend (Figure 1). At 100μM concentration, all drugs except Enrofloxacin, Flumiquine, Sparfloxacin and 8Quinolinol, showed 60-90% inhibition of helicase activity. Similarly, at 10μM concentration, all fluoroquinolones, except Sparfloxacin and 8Quinolol, showed 40-80% inhibition of helicase activity. With the exception of Balofloxacin, Ofloxacin, 8Hydroquinolol, and, Sparfloxacin, all fluoroquinolones showed 30%-60% inhibition of LT-Ag helicase activity at 1 μM (Figure 1).

### Docking studies

**Figure 2:**
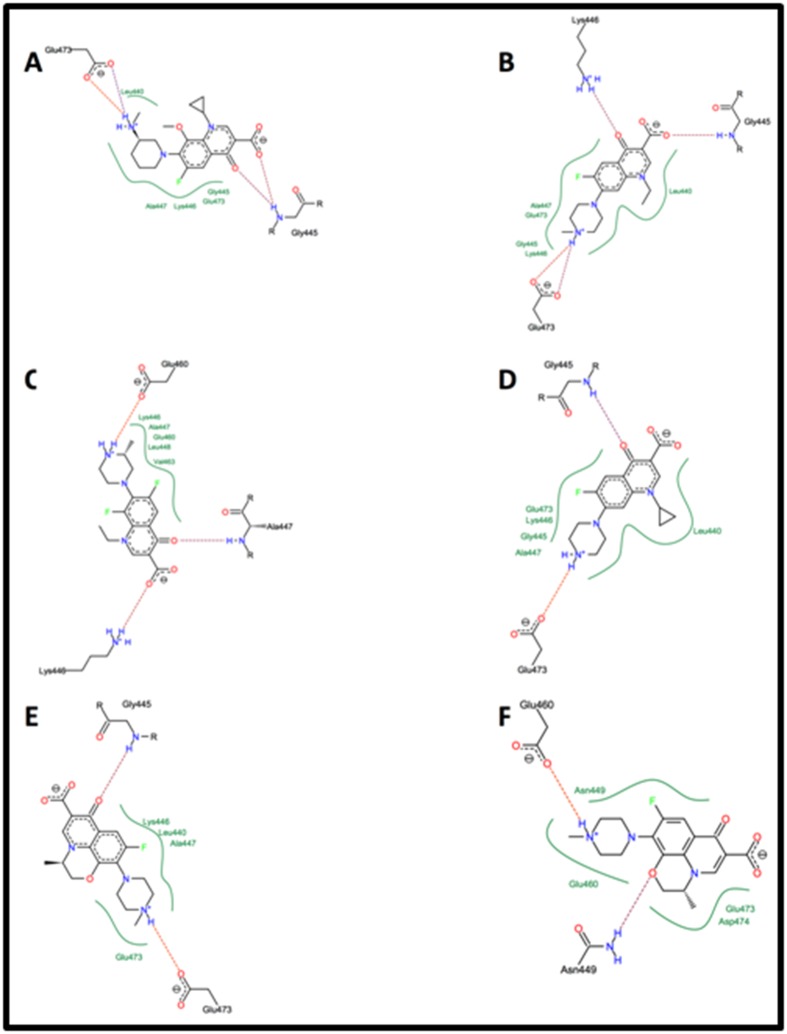
Docked conformation of Fluoroquinolones in the binding cavity of LT-Ag: Docked models in 2D format, generated by FlexX, showing the binding pocket of LT-Ag with docked conformations of (A) Balofloxacin, (B) Pefloxacin, (C) Lomefloxacin, (D) Ciprofloxacin, (E) Levofloxacin, and (F) Ofloxacin. Broken lines indicate H bonding.

The model of SV40 LT-Ag (PDB ID: 1SVO) was docked individually with each of the above-mentioned 18 fluoroquinolones. Each fluoroquinolone was docked deep into the active site of LT-Ag helicase protein molecule. Coordinates of the LT-Ag binding sphere were designated between Glu473 and Lys445. Based on the lowest docking energy, 6 fluoroquinolones, namely, Balofloxacin, Ofloxacin, Pefloxacin, Lomefloxacin, Ciprofloxacin, and Levofloxacin, were selected for detailed analyses of their interactions with LT-Ag (Table 1). Except for Lomefloxacin and Ofloxacin, all six fluoroquinolones showed Hbond interactions with Gly445 and Glu473 in the binding pocket of LT Ag (Table 2 and Figure 2). Although it did not form H-bond interactions, amino acid Leu440 was found in the binding pocket of all fluoroquinolones except Lome- and O-floxacin (Figure 2). Overall, therefore, the interactions made by Lomefloxacin and Ofloxacin were somehow unique compared to other drugs (Table 2 and Figure 2). Based on the free energy values, the affinity of the six drugs for LT-Ag was found to be in the following order: Balofloxacin>Pefloxacin>Lomefloxacin> Ciprofloxacin > Levofloxacin >Ofloxacin (Table 1). The same order was recapitulated when the docking energy of these fluoroquinolones were calculated using a different software, namely, Molegro (Table 1). Overall, Molegro recapitulated the interactions between fluoroquinolones and SV40 LT-Ag (data not shown).

## Discussion

In this study we have used SV40 LT-Ag as a model viral helicase and have explored the inhibitory interactions of fluoroquinoloneson with this enzyme using in vitro as well as in silico approaches. We tested the activity of a panel of 18 fluorquinolones using a helicase assay and found all of them to inhibit the helicase activity of LT-Ag. Docking of these fluoroquinolones on LT-Ag was also performed using in silico methods. Based on the free energy values, 6 fluoroquinolones were found to interact most strongly with LT-Ag, with their binding affinities in the following order: Balofloxacin>Pefloxacin>Lomefloxacin>Ciprofloxacin>Levofloxa cin>Ofloxacin. Docking analysis identified certain amino acids in the LT-Ag active site, namely, Gly445, Glu473, and Leu440 that were found to interact frequently with most of the six selected fluoroquinolones. For this study, we employed the nonradioactive assay that we have previously published [10]. In this assay, 18 different fluoroquinolones were tested on LT Ag helicase activity. All of the tested drugs showed inhibition of helicase activity to some degree. Most fluoroquinolones showed inhibition of 40%-80% at 10μM and 30%-60% at 1μM. In our previously reported study on HCV NS3 helicase, all fluoroquinolones we tested showed inhibition of NS3 helicase activity as well, with Ofloxacin, Fleroxacin, Enoxacin, 8Hydroxyquinolinol, Difloxacin, Flumiquine and 8Quinolinol exhibiting 10-40% inhibition of HCV NS3 helicase at 0.1μM concentration [11]. In another study, helicase activity of SV40 LTAg was inhibited by Levo-, Cipro-, Trovo-, and O-floxacin [8]. The current results are therefore consistent with previous reports.

Although all fluoroquinolones were found inhibitory to both the viral helicases, as expected, the individual fluoroquinolones behaved differently with respect to the level of inhibition observed on HCV NS3 and SV40 LT-Ag. Since the architecture of the helicase active site represents diversification among different viral helicases, the nature of their interaction with fluoroquinolones was expected to differ accordingly. A deeper understanding of the differential interaction of fluororoquinolones on different helicases may be obtained by analyzing crystal structures of helicase-fluoroquinolone complexes and/or by studying the effect of amino acid mutations in the active site of LT-Ag. For the current study, we adopted an in silico approach to further dissect the interactions of fluoroquinolones at the LT-Ag active site.

Computer docking models of fluoroquinolone-LT-Ag helicase also revealed certain key functional groups of fluoroquinolone that were involved in their mutual interaction with LT-Ag helicase. The docked models of fluoroquinolones and LT-Ag showed that the piperazinyl moiety at position 7, carboxylic acid at position 3 and oxoquinoline at position 4 in the fluoroquinolone molecule were mostly involved in interaction with the amino acid residues of LT-Ag. The docking output reflected binding efficiency of LT-Ag and fluoroquinolones. The binding affinity of Balofloxacin was the highest followed by affinities for Pefloxacin, Lomefloxacin, Ciprofloxacin, Levofloxacin, and Ofloxacin (-16.4, -16.1, -15.6, -15.0, -14.0, and - 13.6, kJ/mol, respectively), as predicted by FlexX (Table 1). The same trend was recapitulated when the docking of these drugs was performed using Molegro. All of these fluoroquinolones also showed 60-70% inhibition of helicase activity at 10μM concentration in in vitro helicase assay (Figure 1). The molecular interaction between Ofloxacin and SV40 LT-Ag was found to be weakest as judged by docking energy scores (-13.6), which was in agreement with in vitro assay results, where at 10μM concentration it showed less than 60% inhibition of helicase activity (Figure 1). It may be concluded, therefore, that the energy scores roughly correlated with the performance of fluoroquinolones in the invitro assays. On a deeper level, discrepancies in a drug's in situ and in vitro behavior may also be attributed to the behavior of the drug in solution, and the effects of environmental factors, such as pH and temperature. Interestingly, Ofloxacin and Lomefloxacin showed interactions that were unique from all the other fluoroquinolones we studied. Since, structurally, Ofloxacin and Lomefloxacin are not remarkably different from the rest of the drugs; it is possible that the in situ spatial orientation of these two fluoroquinolones differs from the other drugs we studied. This may be further explored by deeper analysis of docked fluroquinolone-LT-Ag complexes. It would be interesting to design in silico derivatives of fluoroquinolones, combining features of Ofloxacin and Lomefloxacin with other fluoroquinolones, and study their docked complexes with LT-Ag and other helicases. This exercise may provide fluroroquinolone derivatives that inhibit the helicase activity more strongly than the existing drugs.

## Competing Interests

The authors declare that they have no competing interests.

## Figures and Tables

**Table 1:** Docking energies of Fluoroquinolone-LT Ag complexes: Docking energy scores (KJ/mol) werepredicted usingFlexXand Molegrosoftware. The drugs are arranged in ascending order of the docking energy scores of their complexes with SV40 LT-Ag. The energy scores predicted for each drug-protein complex by the two softwares, although on different scales, were in agreement with each other.

Ligand	Docking Energy (KJ/mol)	
FlexX	Molegro
Balofloxacin	-16.4	-73.4
Pefloxacin	-16.1	-68.2
Lomefloxacin	-15.6	-65.7
Ciprofloxacin	-15	-61.4
Levofloxacin	-14	-60.9
Ofloxacin	-13.6	-59

**Table 2:** LT-Ag amino acid residues interacting with Fluoroquinolones: The table shows amino acid residues constituting the binding pocket of LT-Ag. Amino acids shown in bold were found to make H-bond interactions with fluoroquinolones, while the remaining amino acid residues represent residues residing in the binding pocket.

Ligand	Interacting Amino acid Residues
Balofloxacin	Glu473, Gly445, Lys446, Leu440 Ala447
Pefloxacin	Lys446, Gly445 Glu473, Leu440 Ala447
Lomefloxacin	Glu460, Ala447, Lys446, Leu448 Val463
Ciprofloxacin	Gly445, Glu473, Leu440, Ala447, Lys446
Levofloxacin	Gly445, Glu473, Ala447, Leu440, Lys446
Ofloxacin	Glu460, Asn449, Glu473, Asp474
